# The potential effect of albumin replacement on immune modulation and sphingosine 1-phosphate dynamics

**DOI:** 10.1038/s41598-026-38157-8

**Published:** 2026-02-06

**Authors:** M. S. Winkler, F. Enzmann, M. Schilder, I. Seidita, O. Moerer, M. H. Gräler

**Affiliations:** 1 Department of Anesthesiology, University Medical Center Göttingen, Robert-Koch- Str. 40, 37075 Göttingen, Germany; 2 Department of Anesthesiology and Intensive Care Medicine, Center for Molecular Biomedicine (CMB), Jena University Hospital, Hans-Knöll-Str. 2, 07745 Jena, Germany; 3 Center for Sepsis Control and Care, Jena University Hospital, 07740 Jena, Germany; 4 Present Address: Department of Experimental and Clinical Biomedical Sciences “Mario Serio”, University of Florence, Viale Morgagni 50, Florence, 50134 Italy

**Keywords:** Albumin, Sphingosine 1-phosphate (S1P), High-density lipoprotein (HDL), Lymphocytes, Barrier function, Biochemistry, Immunology

## Abstract

Hypoalbuminemia is common in critically ill patients, yet albumin replacement remains controversial. Beyond volume regulation, albumin transports sphingosine 1-phosphate (S1P), a key modulator of immunity and endothelial stability. This study investigates how human albumin (HA) affects S1P distribution between high-density lipoprotein (HDL-cholesterol, HDL) and serum albumin (SA) and its impact on lymphocyte migration and vascular integrity ex vivo. Between March 2022 and February 2023, a prospective observational cohort study (AlbuS1P Study) was conducted in an intensive care unit (ICU). A total of 47 patients were enrolled and stratified based on baseline SA levels: Group A (normal SA, untreated control), Group B (low SA, untreated control) and Group C (low SA, treated with 180 g HA over three days). Blood samples were collected at multiple time points for laboratory analyses, including S1P quantification, immune cell assessments, ApoM ELISA and flow-induced dispersion analysis (FIDA) to determine S1P binding to SA or HDL. In this cohort study of 42 ICU patients with hypoalbuminemia, HA administration increased SA-levels (+ 0.7 g/dL, + 40%) but had no effect on free plasma S1P levels. HA treatment tended to shift S1P binding from HDL to SA in vivo and coincided with lower CD4⁺ T cell and CD19⁺ B cell counts, a finding that could be consistent with, but does not prove, reduced S1P-driven immune cell migration. In contrast, ex vivo assays showed no significant effects of HA-treated plasma on endothelial barrier function or S1P-mediated lymphocyte chemotaxis. These findings are consistent with the notion that HA treatment may modulate S1P distribution in vivo in patients, potentially influencing immune cell dynamics without evidence of impaired vascular stability. HA redistributed S1P from HDL to SA without altering total plasma S1P levels. This shift may correlate with immune modulation and had no negative effect on endothelial stability or cell migration ex vivo. Future studies should identify patient endotypes and subgroups, such as those with low HDL or hyperinflammation, who may benefit from HA’s immunomodulatory effects.

## Introduction

Hypoalbuminemia is a common phenomenon in critically ill patients and is associated with increased mortality^1–4^. However, the replacement of human albumin (HA) remains controversial. A 2011 Cochrane analysis (38 studies, > 10,000 patients) showed no impact on mortality (RR 1.02; 95% CI 0.92–1.13), though outcomes varied by disease - e.g., a nearly threefold increased risk in burn patients^5^. Recommendations for HA in sepsis have been partially revised due to methodological concerns in some studies^6^, further increasing uncertainty. Three key trials - EARSS (1998), SAFE (2004), and ALBIOS (2014) – found no significant mortality benefit of HA in sepsis^7–10^. However, a predefined subgroup analysis of the ALBIOS trial demonstrated a 12.6% relative reduction in mortality among patients with septic shock receiving albumin, suggesting that albumin may exert context-dependent biological effects in specific patient subgroups^11^. Accordingly, current guidelines remain inconsistent: HA is only weakly recommended, particularly in cases of high crystalloid demand and experts are still uncertain^12–14^. The discrepancy between clinical evidence and medical practice continues to be investigated^15,16^. Albumin contributes 60% to oncotic pressure, regulates volume status, and may play a more significant role in immune modulation than in simple protein replacement^17,18^. If confirmed, this would necessitate an individualized approach to HA administration based on the patient’s inflammatory status rather than just volume deficiency^19^.

A key factor in these non-oncotic effects is sphingosine 1-phosphate (S1P), which is transported by albumin. Low S1P levels correlate with disease progression and mortality in sepsis^20^ and influence two critical processes: lymphocyte migration (S1P deficiency leads to lymphopenia, as seen in Gilenya^®^ therapy^21,22^) and endothelial barrier stabilization, which may prevent sepsis-induced vascular leakage^23^.

Since S1P is mainly bound to the endogenous carrier molecules serum albumin (SA) and high-density lipoprotein (HDL, determined by HDL-cholesterol) in blood^24^, HA administration may influence its bioavailability and function. This prospective cohort study will be the first to investigate the effect of HA intervention on S1P compartments (HDL, SA) and their impact on lymphocyte migration and endothelial stabilization ex vivo.

## Materials and methods

### Patients and study design

Between March 2022 and February 2023, we conducted an observational, prospective cohort study to assess changes in S1P under HA treatment. The *AlbuS1P Study* (*“Non-oncotic effects of albumin as a carrier of sphingosine-1-phosphate (S1P) for the restoration of endothelial cell barriers and immune cell function in sepsis”*) was reviewed by the Ethics Committee of University Medicine Göttingen (AlbuS1P 7/11/21) and registered in the local trial center (2020 − 01705 UMG) and the German Registry for Clinical Studies (DRKS00027834). All research involving human participants was approved by University Medical Center Göttingen, and conducted in accordance with relevant guidelines and regulations, including the Declaration of Helsinki. Informed consent was obtained from all participants and/or their legal guardians prior to participation. A total of 47 adult patients from our anesthesiological intensive care unit were enrolled after obtaining written informed consent. Based on their SA levels, patients were divided into three groups: (A) ICU controls with normal SA levels of 3.0 to 5.0 [g/dL], (B) untreated ICU controls with low SA levels (< 3 g/dL), and (C) a treatment group with low SA levels (< 3 g/dL) that received a total of 180 g HA (administered as three daily doses of 20% HA for three consecutive days). The non-interventional study left HA administration entirely at the discretion of attending physicians, independent of the study. Treating physicians had no access to study results, and patient inclusion did not alter therapy. For patients receiving HA, laboratory measurements were taken at study inclusion (day 0), and on days 1, 2, and 3. HA administration followed the hospital’s standard operating procedure (SOP).

### Clinical evaluations

Blood samples (EDTA & Serum) were collected from patients in the treatment group on four separate days, whereas in the other groups, only one sample was taken. The first blood sample in the HA treatment group was collected prior to the administration of HA. On each study day, the Institute of Clinical Chemistry at the University Hospital Göttingen measured a range of standard and extended laboratory parameters. These included lipoproteins such as HDL and LDL (Low-Density Lipoprotein, determined by their respective cholesterol content HDL-C and LDL-C), total cholesterol, triglycerides, albumin, liver enzymes, creatinine, platelets, C-reactive protein (CRP) and a differential blood count. Additionally, lymphocyte subpopulations were assessed by fluorescence-activated cell sorting (FACS), allowing for a detailed analysis of the immune cell populations.

### Processing

Blood samples were stored upright at room temperature for 30 min immediately after collection to allow for clotting. They were then centrifuged at 2500 g for 10 min at 20 °C to separate the plasma from cellular components. The supernatant was transferred to a 2 ml container and frozen at -20 °C for later analysis. These frozen samples were subsequently processed further at the University Hospital Jena to analyze the relevant laboratory parameters and to conduct the desired evaluations.

### S1P determination

Sphingosine-1-phosphate (S1P) levels in blood were measured in single measurements using high-performance liquid chromatography (HPLC) coupled with a triple-quadrupole mass spectrometer (LC/MS/MS). C17-S1P (100 pmol/sample) was added as an internal standard. After adding methanol and chloroform, the lipids were extracted twice by vortex mixing and centrifugation. The chloroform phase, which contained the lipids, was transferred for further analysis. After drying the samples at 60 °C under vacuum, the lipids were resuspended in a methanol/chloroform mixture and stored for further analysis. The S1P and C17-S1P concentrations were quantified using positive electrospray ionization with multiple reaction monitoring (MRM) transitions (S1P m/z 380/264 and C17-S1P m/z 366/250) on a triple-quadrupole mass spectrometer. Chromatographic separation was performed on a C18 column, and data were analyzed through linear regression to determine the concentrations. We described this method in our previous publications in detail^20^.

### Apolipoprotein M (ApoM) quantification

Plasma ApoM was measured by ELISA according to the manufacturer’s instructions (MAPTECH, Cincinnati, OH, USA; single measurements; standard curve R^2^ = 0.995; reportable range per kit specification). Values are reported as concentration in plasma.

### FIDA (Flow-induced dispersion analysis)

Flow-induced dispersion analysis (FIDA) was performed using the FIDA-1 system to measure the hydrodynamic radius and to detect S1P complex formation with HDL and SA. S1P-fluorescein (S1P-FITC) was used as the indicator. The S1P concentration was diluted to 50 nM or 500 nM in PBS (phosphate-buffered saline), and 15 µl of plasma samples were also diluted to 10% in PBS. The analysis was carried out in triplicates by capillary flow at 37 °C under standardized conditions, with the flow properties of the sample being monitored in real time via a fluorescence detector. The capillary analysis allowed precise characterization of the complex formation between S1P and HDL or SA. The results were analyzed using the FIDA software V2.32. The detailed method was recently described and reported by our group^25^.

### Transwell chemotaxis assay

Lymphocyte migration was assessed in single measurements using a Transwell chemotaxis assay with 24-well plates, where culture inserts with polycarbonate filters (6.5 mm diameter, 5 μm pore size) were pre-coated with human collagen type IV. The inserts were placed in new plates and washed with PBS before being air-dried under sterile conditions. The lower chambers of the new plates were filled with 10% patient samples, and the prepared inserts were placed into the wells. For the migration assay, Ramos cells expressing enhanced green fluorescent protein (eGFP) for S1PR1 were used. The cells were kindly provided by Hermann Eibel (Medical Center of the University of Freiburg, Germany). Each insert was seeded with 500,000 cells, incubated for 3 h at 37 °C, and then removed. Cells that migrated into the lower chamber were quantified using FACS (fluorescence-activated cell sorting). The data were processed using the Accuri C6 Plus software V1.0.23.1 to assess migration and response of the lymphocytes to S1P stimulation.

### ECIS (Electric cell-substrate impedance sensing)

Electric cell-substrate impedance sensing (ECIS) was used to assess the impact of patient samples on the barrier function of endothelial cells. EA.hy926 cells (RRID: CVCL_3901) were cultured on ECIS arrays, which were pre-coated with gelatin. Once a confluent monolayer formed, the growth medium was replaced with serum-starvation medium (2% FBS). After six hours, the cells were stimulated with 10% patient samples, and impedance measurements were continuously recorded to monitor changes in the cell barrier function. The impedance data were analyzed in single measuements using ECIS software V1.4.18.0, and the barrier function was determined by comparing the impedance values before and after stimulation.

### Calcium signaling at S1PR1 and S1PR3

To compare carrier-specific S1P effects, we recorded S1P-evoked Ca²⁺ responses in HTC4 cells (RRID: CVCL_D053) overexpressing S1P receptor type 1 or type 3 (S1PR1, S1PR3). Cells were loaded with FURA-2/AM (MoBiTec, Göttingen, Germany) and stimulated with S1P at low nanomolar concentrations (1nM), either alone or pre-complexed with physiological plasma concentrations of human HDL (0.5 mg/mL) or HA (50 mg/mL). Responses were normalized to an ATP reference pulse (10 µM) and expressed as % of ATP, *n* = 5 independent experiments.

### Statistical analysis

Statistical analyses were performed using GraphPad Prism version 7.0a (La Jolla, CA, USA) and SPSS Statistics version 21 (IBM Corporation, Armonk, NY, USA). Group differences were assessed using the non-parametric Mann–Whitney U test or Kruskal–Wallis analysis of variance, and categorical variables, including mortality, were compared using Fisher’s exact test. Multivariable linear regression analyses were applied to evaluate associations of serum albumin-bound (SA-S1P) and HDL-bound (HDL-S1P) sphingosine 1-phosphate with relevant outcomes, adjusting for age, sex, and baseline disease severity. Outliers were not removed, and no formal correction for multiple comparisons was applied due to the exploratory nature and limited sample size of the cohort. Data are presented as median [interquartile range, IQR], and two-sided P values < 0.05 were considered statistically significant.

## Results

### Low HDL levels are associated with HA administration

Between 03/2022 and 02/2023, a total of 47 ICU patients were included in the study. All patients, whether treated with HA or not, met the inclusion criterion of hypoalbuminemia (Table 1). Median SA levels were similar between the ICU treated (1.8 g/dL) and ICU untreated groups (2.0 g/dL) but significantly lower than in ICU controls or group A (3.3 g/dL). The decision to administer HA was made independently by the attending physicians. Twenty-two patients received a cumulative dose of 180 g HA over three days (9 × 100 mL of 20% HA), while 20 patients with equally low SA levels did not receive HA. This difference was likely due to varying disease severity, as SOFA scores were significantly higher in the treatment group C (Table 1). Baseline disease severity differed between treatment groups: HA recipients had higher SOFA at inclusion (median 9 versus 4, see Table 1), whereas SAPS II was not significantly different (median 40 versus 33, see Table 1). Although mortality did not differ significantly between the groups, patients in the HA-treated group tended to have a longer ICU stay and a numerically higher mortality. In the untreated group, three patients died - one from pulmonary embolism, one after a traumatic fall during mobilization, and one following therapy withdrawal after prolonged ECMO support. In contrast, among the eight deaths in the treatment group, the majority were directly related to infectious complications, including six due to septic shock and one due to pancreatitis with secondary infection; one additional patient died following therapy withdrawal.

**Fig. 1:**
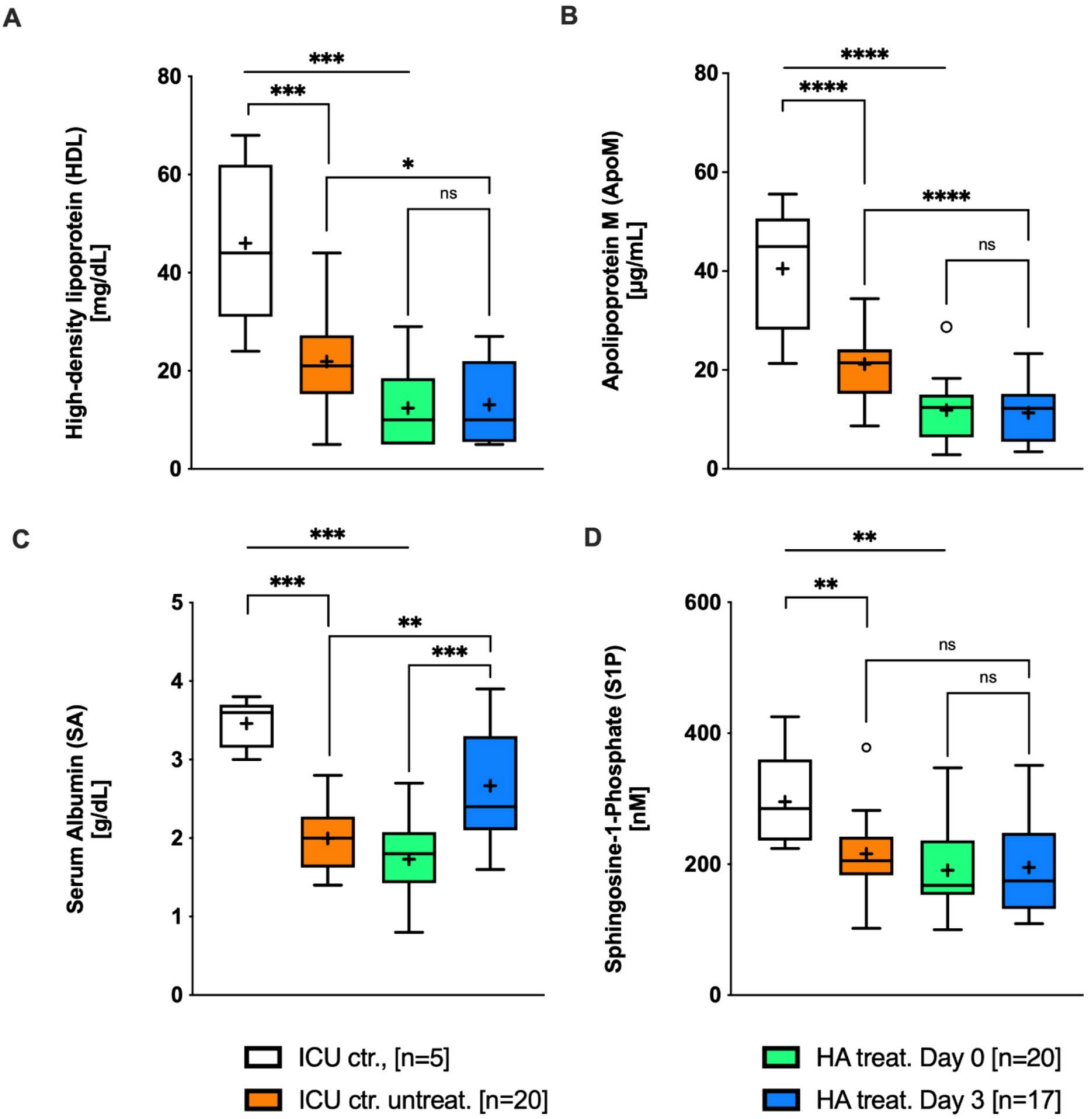
Overview of patient sphingosine 1-phosphate (S1P), serum albumin (SA), high-density lipoprotein (HDL-cholesterol, HDL), and apolipoprotein M (ApoM) levels in all groups and within the human albumin (HA)–treated patients at inclusion (day 0) and after treatment (day 3). Based on their serum albumin (SA) levels, patients were divided into three groups: ICU controls with normal SA levels (3–5 g/dL), ICU controls with low SA levels (≤ 3 g/dL) who remained untreated, and an HA-treated group with low SA levels (≤ 3 g/dL) that received a total of 180 g HA (administered as 100 mL of 20% HA three times daily for three consecutive days). (**A**) HDL and (**B**) ApoM levels were measured and analyzed in the same manner. ICU patients with low SA levels also showed significantly lower HDL levels compared with ICU controls with normal SA levels. HA supplementation had no effect on HDL concentrations. (**C**) SA levels were measured in all groups. ANOVA revealed significant differences between the three groups. HA substitution resulted in a significant increase (Δ) in SA levels during the observation period. (**D**) Plasma S1P concentrations were significantly higher in ICU controls with normal SA levels compared with both, ICU patients with low SA levels who remained untreated and those in the HA-treated group. However, HA substitution did not lead to changes in S1P concentrations during the treatment period. **P < 0.01; *** *P* < 0.001; **** *P* < 0.0001. Statistical analysis: Kruskal–Wallis ANOVA for comparisons between ICU controls with normal SA levels, ICU controls with low SA levels, and HA-treated groups at day 0 (inclusion); Mann–Whitney test for two-group comparisons. Data are shown as box-and-whisker plots with mean (+).

Interestingly, while there were no differences in immune cell populations at baseline, a notable association was observed: patients receiving HA had the lowest HDL levels compared to untreated patients, group B, or ICU controls, group A. However, HDL levels were not a formal criterion for the decision to administer HA (Table 1; Fig. 1A). Similarly, and in line with the HDL concentrations within the study population, ApoM concentrations were also reduced in ICU patients compared with both control groups (Table 1; Fig. 1B). Thus, ApoM concentrations followed a similar pattern as HDL concentrations. In the HA-treated subgroup, ApoM concentrations remained stable from day 0 to day 3 (Fig. 1B).

**Table 1:** Overview of baseline parameters in all studied groups.

Parameter	Reference	ICU ctr.	ICU ctr. untreat.	HA treat. (Day 0, inclusion)	*P*-Value^#^ (A) to (C)	*P*-Value^##^ (B) vs. (C)
Group definition	N/A	(A) Normal SA levels	(B) Low SA levels	(C) Low SA levels	N/A	N/A
Patients, n	N/A	5	20	22	N/A	N/A
ICU mortality, n (%)	N/A	0	3 (0.15)	8 (0.36)	ns^++^	ns^++^
Serum-Albumin (SA)	3.0–5.0 [g/dL]	3.3 (2.9–3.7)	2.0 (1.6–2.3)	1.8 (1.4–2.1)	< 0.001	ns
High-density lipoprotein (HDL)	> 45 [mg/dL]	44 (31–62)	21 (15–27)	10 (5–18)	< 0.05	< 0.01
Apolipoprotein M (ApoM)	N/A	45 (28–51)	21 (15–24)	12 (6–15)	< 0.0001	< 0.001
Sphingosine 1-Phosphate (S1P)	N/A	285 (236–360)	205 (183–242)	167 (153–236)	< 0.01	ns
Age	N/A	46 (34–72)	61 (50–76)	61 (50–70)	ns	ns
Gender, male/female [n]	N/A	4/1	10/6	14/8	N/A	N/A
BMI	18.5–24.9	24.9 (20.1–26.1)	27.3 (22.9–30.0)	24.55 (22.1–28.6)	ns	ns
LOS, days	N/A	7 (2–12)	14 (4–24)	20 (9–32)	< 0.05	ns
SOFA	N/A	2 (0–4)	4 (2–7)	10 (6–13)	< 0.05	< 0.01
SAPS II	N/A	21 (13–28)	33 (24–47)	40 (31–51)	< 0.05	ns
C-reactive protein (CRP)	< 5 [mg/dL]	49 (15–166)	105 (68–154)	172 (73–275)	ns	ns
Leucocytes	4.0–11.0 [x10^3^µL]	9.7 (8.1–10.2)	10.0 (8.0–15.0)	11.6 (7.1–15.5)	ns	ns
Lymphocytes, % (FACS)	20–45 [%]	7 (5–18)	11 (7–15)	10 (3–18)	ns	ns
Total CD3 + T-cells	700–2100 [cells/µL]	365 (235–1402)	694 (550–1210)	679 (297–1141)	ns	ns
CD3+/CD4 + T-helper	300–1400 [cells/µL]	326 (149–910)	475 (303–737)	422 (203–759)	ns	ns
NK-cells	90–600 [cells/µL]	64 (50–176)	90 (69–112)	34 (22–97)	ns	ns
CD19 + B-cells	100–500 [cells/µL]	87 (70–116)	89 (54–175)	94 (57–196)	ns	ns
CD3+/ CD8 + T-Suppressor	200–900 [cells/µL]	99 (50–545)	222 (93–345)	138 (50–384)	ns	ns

BMI, Body mass index; LOS, length of stay in ICU; SOFA, Sequential organ failure assessment score; SAPS II, Simplified Acute Physiology Score II, FACS, fluorescence-activated cell sorting; N/A, not applicable; ns, not significant; Median and interquartile range (IQR); ^#^P-Value determined by ANOVA Kruskal-Wallis for comparison of group A-C. ^##^Pairwise ICU ctr. untreat. versus HA treat.(Day 0, inclusion) comparisons by Mann–Whitney.++Fisher-Exact-test for mortality comparison.

### HA increases serum albumin but does not affect free plasma S1P levels

HA administration significantly increased SA levels over the treatment period (Fig. 1C). A total dose of 180 g HA led to a marked rise in SA concentrations, confirming the expected association between HA infusion and SA increase. This demonstrates the intervention’s effectiveness. However, HA had no direct effect on plasma S1P levels. S1P concentrations were significantly lower in both untreated and treated patients compared to ICU controls and remained unchanged despite HA administration (Table 1; Fig. 1D). Even after three days, S1P levels remained at baseline (Fig. 1D).

### HA does not affect free plasma S1P but alters its binding to HDL

**Fig. 2:**
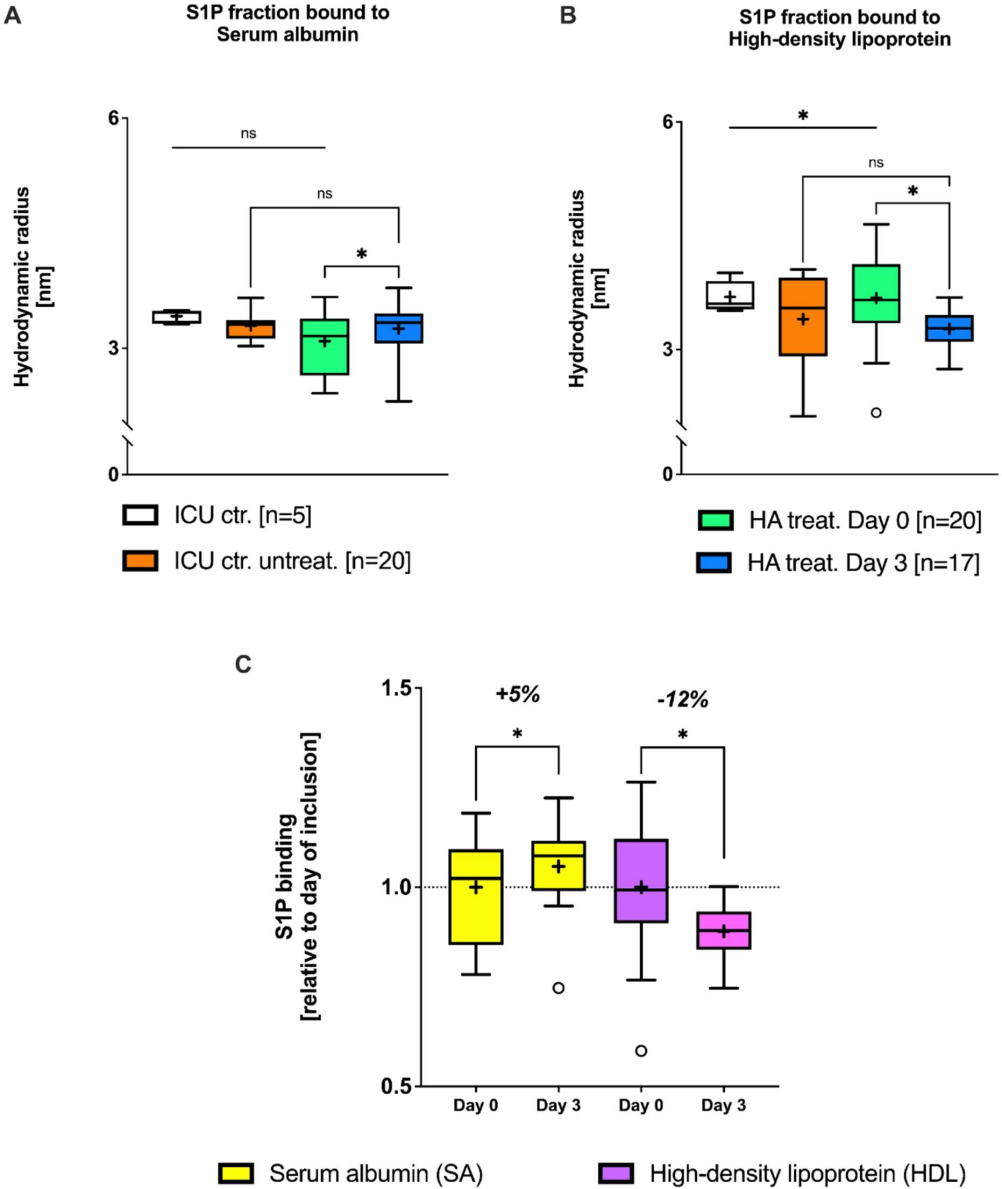
S1P Fractions Determined by flow-induced dispersion analysis. (**A**) S1P fraction bound to serum albumin levels within the groups: In the treatment group with human albumin (HA), the S1P fraction bound to serum albumin increases slightly but significantly. (**B**) S1P fraction bound to high-density lipoprotein (HDL-cholesterol, HDL): Under HA substitution, there is a significant reduction in the S1P fraction compared to both the control and treatment conditions. (**C**) Relative representation of the relationship from Fig. 3A and B: While HA supplementation leads to a slight increase in the S1P fraction in serum albumin, it results in a significant decrease in HDL-bound S1P. *P < 0.05. Mann-Whitney test for two-group comparison. Box-whisker plot with mean (+).

Using FIDA, we examined changes in fluorescein-labelled S1P binding to SA and HDL by determining the hydrodynamic radii of the respective SA-S1P and HDL-S1P complexes as a measure of their size (Fig. 2). At baseline, there were no significant differences between ICU controls (A), untreated (B), and treated patients (C). The mean hydrodynamic radii ranged between 3.1 nm and 3.3 nm for SA-S1P and 3.4 nm and 3.7 nm for HDL-S1P across all groups (Fig. 2). Following HA administration, there was a significant increase in SA-S1P complex size, indicating increased binding of S1P to SA (Fig. 2A). Concomitantly, the complex size of HDL-S1P decreased considerably, even falling below ICU control levels, which pointed to decreased binding of S1P to HDL (Fig. 2B). After normalization of the correspondent complex sizes of SA-S1P and HDL-S1P, HA treatment led to a 5% increase in the SA-S1P complex size and a 12% reduction in the HDL-S1P complex size, clearly demonstrating a shift of S1P binding from HDL to SA (Fig. 2C).

Given that the clinical decision to initiate HA may introduce treatment selection bias, statistical analyses primarily focused on within-patient changes (Δ day 0–3) in the HA-treated group. To account for potential baseline imbalances, multivariable linear regression analyses were performed including baseline SOFA, age, and sex as covariates. For SA-S1P, the unadjusted paired change was + 0.162 (95% CI 0.031–0.294); after adjustment, the direction and magnitude remained unchanged, and none of the covariates (SOFA, age, sex) were significant (SOFA β = +0.014, 95% CI − 0.026 to 0.053, *P* = 0.46). For HDL-S1P, the unadjusted paired change was − 0.408 (95% CI − 0.724 to − 0.092). In the adjusted model, higher baseline SOFA tended to predict a larger decrease (β = −0.065 per SOFA point, 95% CI − 0.133 to 0.003, *P* = 0.06), and age showed a modest positive association (β = +0.018 per year, 95% CI 0.001 to 0.035, *P* = 0.04), whereas sex was not significant (data not presented in a table).

### Potential effects on immune cell migration

**Fig. 3:**
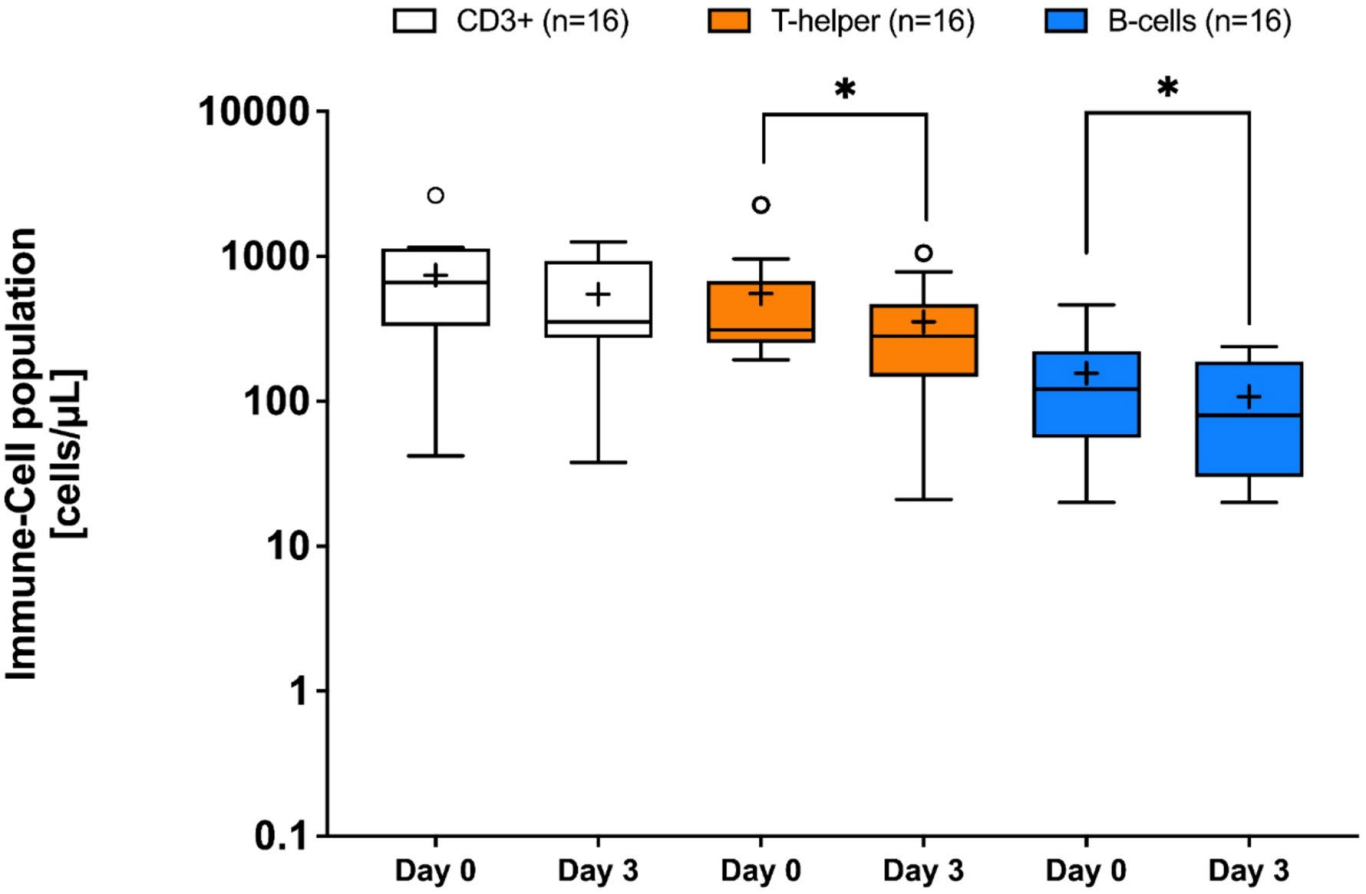
Overview of Immune Cell Population in cells/µL in sixteen human albumin treated ICU patients The figure shows that the immune cell population changes under treatment with human albumin (HA). Over the three-day treatment period, fewer CD3+/CD4 + T-cells and CD19 + B-cells are present in peripheral blood compared to baseline. *P < 0.05, Mann-Whitney test for two-group comparison. Box-whisker plot with mean (+).

S1P is crucial for lymphocyte homing, and blocking S1P receptor type 1 (S1PR1) can completely inhibit this migration. It is unclear whether this effect can be modulated by S1P carrier molecules such as SA or HDL. The analysis of T and B cell counts in blood before and 3 days after treatment with HA demonstrated a significant reduction of CD4-positive T cells and CD19-positive B cells (Fig. 3). It is an interesting hypothesis that HA administration and the resulting redistribution of S1P from HDL to SA may be associated with reduced S1P-driven immune cell migration into peripheral blood. (Fig. 3).

### Plasma barrier-stabilizing capacity and lymphocyte chemotaxis were comparable across groups and unaffected by HA

**Fig. 4:**
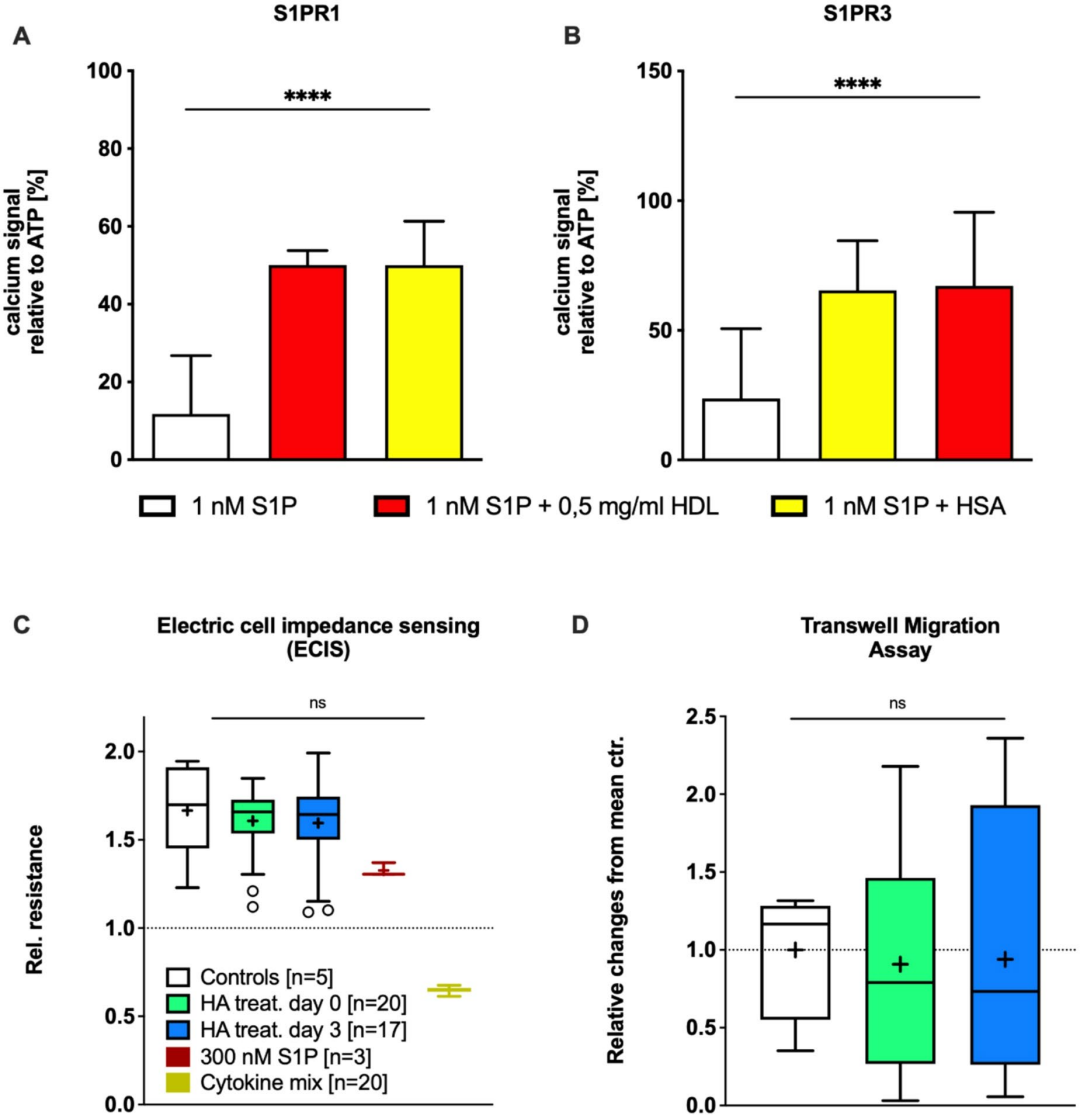
In vitro data on Ca²⁺ signaling and functional ex-vivo assays. (**A**, **B**) Ca²⁺ signaling in S1PR1- and S1PR3-overexpressing cells. The S1P-dependent Ca²⁺ response was enhanced by S1P carrier proteins, either human albumin (HA) or high-density lipoprotein cholesterol (HDL). Data were normalized to the mean response of the S1P-only condition, *n* = 5 independent experiments. (**C**) Endothelial barrier stabilization assessed by electric cell-substrate impedance sensing (ECIS). Resistance was normalized to baseline values before stimulation (dotted line). 300 nM S1P dissolved in 50 mg/mL HA was used as positive control. As a negative control, the endothelial barrier was disrupted with a mix of LPS and cytokines (50 ng/mL IL1β, 50 ng/mL TNFα, 1 µg/mL LPS). Supplementation with HA had no direct effect on endothelial barrier stabilization. (**D**) Immune cell migration analyzed in a Transwell assay. Supplementation with HA did not negatively affect lymphocyte migration. Data were normalized to the mean of the ICU control group. *P < 0.05, Kruskal–Wallis ANOVA for group comparisons. Data are presented as box–whisker plots with mean (+).

Carrier-defined Ca²⁺ signaling indicated comparable potency at S1PR1 and S1PR3. In Ca²⁺ assays using matched low nanomolar S1P concentrations, both HDL-S1P and HA-S1P enhanced the S1P-evoked Ca²⁺ signal compared with S1P alone, without differences in peak amplitude between carriers (Fig. 4A,B). As HDL-bound S1P has previously been implicated in endothelial barrier stabilization^26,27^, we next examined whether the observed shift from HDL-S1P to SA-S1P following HA administration affected endothelial integrity. In electric cell–substrate impedance sensing (ECIS) measurements using Ea.hy926 endothelial cells, plasma from HA-treated patients showed no reduction in impedance compared with controls after HA administration (Fig. 4C), indicating that endothelial barrier function was preserved. To assess potential immunological consequences, S1P-dependent chemotaxis was analyzed using S1PR1-overexpressing RAMOS B cells exposed to patient plasma. Plasma from HA-treated patients exhibited slightly lower baseline migratory activity, which remained unchanged following HA administration (Fig. 4D). This difference was not statistically significant. Together, these data indicate that HA treatment does not impair endothelial barrier stabilization or S1P-driven B cell chemotaxis ex vivo, despite the carrier redistribution of S1P observed in vivo .

**Fig. 5:**
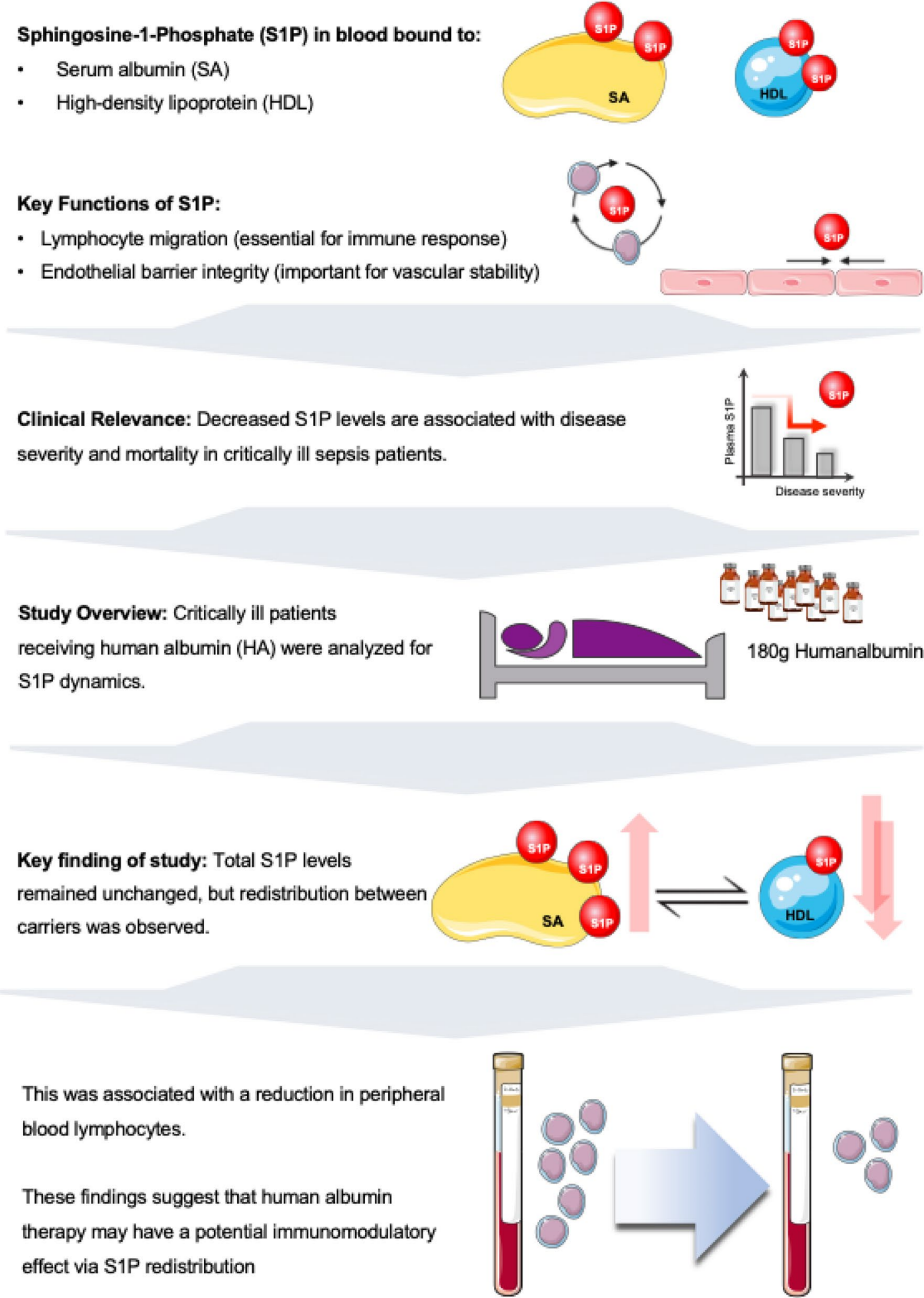
Summary of study design, key findings, and mechanistic hypothesis. This figure illustrates the study design and main observations. S1P in blood is bound to SA and HDL and plays key roles in lymphocyte migration and endothelial barrier integrity. In critically ill patients, decreased S1P levels are associated with disease severity and mortality. Our study shows that HA treatment redistributes S1P between carrier molecules without affecting total plasma levels, and this redistribution correlates with reduced peripheral blood lymphocytes. We propose the hypothesis that this S1P redistribution may mediate potential immunomodulatory effects of albumin therapy, which warrants further mechanistic investigation.

## Discussion

Our data show that HA substitution successfully corrects clinically measurable hypoalbuminemia but does not affect total plasma S1P concentration. This study demonstrates that HA administration in critically ill patients leads to S1P redistribution between carrier molecules (Fig. 5), which may have immunomodulatory implications.

The observation of reduced plasma S1P levels in patients, regardless of treatment, is consistent with numerous previous clinical studies^28–32^. This supports the assumption that S1P levels are significantly reduced in critically ill patients - a condition likely associated with the frequent protein deficiency seen in ICU patients. One of our hypotheses was that compensating for albumin deficiency would lead to an increase in total plasma S1P levels. This would be plausible since S1P is primarily stored in erythrocytes, and albumin promotes its release while also regulating microvascular permeability^33,34^. Nevertheless, S1P levels – already reduced compared to the control group – remain unchanged under treatment, even after a cumulative dose of 180 g HA (Figs. 1 and 2). This observation is consistent with a recently published follow-up of the ALBIOS trial^35^, which investigated 375 patients with septic shock. The median S1P concentration of 86 [ng/mL] or 226 [nM] found in that study is in the same range of our measurements of 170–200 [nM] (Fig. 1C). The marginal median S1P increase of only 6 [ng/mL] due to the intervention is also reflected in our results (Fig. 1C).

This could suggest that HA has no influence on biologically active S1P signalling. However, the discrepancy remains between the belief in its therapeutic benefits and the evidence regarding S1P-dependent signaling pathways. A survival benefit of HA treatment, though not statistically significant, becomes more pronounced with increasing disease severity^10,11^. Consistent with this, the ALBIOS trial observed that S1P levels in septic shock were inversely proportional to markers of endothelial dysfunction and coagulation: Patients with low S1P levels exhibited significantly elevated plasma levels of syndecan-1 (Syn-1)^35^. While this study did not specifically investigate S1P-binding proteins, our research group used flow-induced dispersion analysis (FIDA) to show that particularly low HDL-S1P levels are associated with disease severity, such as the need for mechanical ventilation^25^. The question of what happens to S1P fractions when HA is substituted had previously remained unanswered.

Since S1P circulates bound to specific carrier proteins, changes in these carriers can shift the equilibrium of S1P distribution. This hypothesis is indeed confirmed by our data (Fig. 2): while HA administration compensates for hypoalbuminemia, the SA-S1P fraction increases and the HDL-S1P fraction decreases. This change is molecularly plausible. S1P release is an active, ATP-dependent process mediated by the transporters Mfsd2b in erythrocytes and Spns2 in other tissues^36,37^. Albumin regulates S1P release via Mfsd2b^33^, whereas HDL—particularly its ApoM component—interacts with Spns2 in endothelial cells^38^. This aligns with our observation that HDL and ApoM levels change in parallel and are inversely related to disease severity (Fig. 1A,B).

The stability of S1P binding in plasma depends on the molar concentration of its carriers. Under physiological conditions, ApoM(+)HDL and serum albumin exhibit comparable affinities for S1P^38^, which may explain the observed redistribution when one carrier fraction markedly increases. Additionally, the half-life of SA-S1P is shorter than that of HDL-S1P, potentially leading to HA-induced release and accelerated degradation^34^. Ex-vivo studies have further shown that ApoM(+)HDL-S1P elicits stronger S1PR1/S1PR3-mediated signaling than SA-S1P, while no difference was detected for ERK pathway activation^38^. Consistently, both carriers enhanced S1P-induced Ca²⁺ signaling under low-nanomolar conditions (Fig. 4A,C).

The importance of HDL for S1P-dependent functions is further supported by studies in PLTP-deficient mice, which exhibit markedly reduced S1P levels—not because PLTP binds S1P directly, but because it regulates HDL metabolism and thereby S1P bioavailability^39^. The same study showed that albumin deficiency had no impact on total plasma S1P, reinforcing that HDL, rather than albumin, is the key determinant of circulating S1P levels (Fig. 1), consistent with previous observations for ApoM(+)HDL^30,40^. ApoM(+)HDL may thus serve as a biomarker of disease severity in sepsis, as experimental studies have demonstrated its endothelial-protective effects^41^. Loss of ApoM(+)HDL-S1P impairs S1PR1 signaling and destabilizes the endothelial barrier^42^. Indeed, endothelial protection is largely mediated by HDL-bound S1P, and depletion of its carrier ApoM(+)HDL is associated with inflammatory processes^43,44^, whereas albumin-bound S1P may regulate other physiological functions^34^. Collectively, these findings provide a conceptual illustration of the complex mechanistic interdependence between the biological effects of S1P, its carriers, and their target structures.

S1P- and carrier-dependent effects across different cell types and tissue compartments may therefore underlie both our observations and prior findings in the literature. Future studies should investigate S1P-mediated signaling across compartments and delineate carrier-specific pathways in greater mechanistic detail. However, our results are unlikely to be explained by a single mechanism, as no direct ex vivo effects were observed. Additional processes - such as apoptosis or altered immune cell turnover - are also likely to contribute in the complex pathophysiological context of critical illness.

Previous work has suggested carrier-dependent differences in S1P signaling kinetics, for example, sustained S1PR1/S1PR3 activation by ApoM(+)HDL-S1P. In contrast, our Ca²⁺ amplitude measurements under matched low-nanomolar conditions showed similar enhancement for HDL-S1P and SA-S1P. This likely reflects assay-specific factors (receptor background, ApoM content of HDL, concentration range) and the limitation of amplitude-based readouts, which do not capture kinetic or downstream pathway differences. Accordingly, we avoided overinterpretation of qualitative carrier effects and focused on the key observation that HA-induced S1P redistribution did not translate into measurable functional impairment in our ex vivo assays.

Our ApoM analyses further indicate that the HA-induced shift of S1P away from HDL is unlikely to result from ApoM depletion. Rather, the data are consistent with a concentration-dependent equilibrium effect driven by the marked rise in serum albumin, which increases the available binding capacity for S1P. Although ApoM(+)HDL remains mechanistically important, ApoM levels per se did not decline during HA administration in this cohort.

Finally, we observed alterations in immune cell populations (Table 1), particularly within the T- and B-cell lineages (Fig. 3). Although no direct effects on B-cell migration or endothelial barrier stabilization were detected ex-vivo (Fig. 4), these findings may suggest that S1P-HDL modulates lymphopoiesis or immune cell homeostasis rather than migratory behavior^44^.

### Limitations

#### Study design and patient heterogeneity

This was a single-centre, observational cohort in which human albumin (HA) was administered according to clinical judgement. Although all participants exhibited hypoalbuminemia, the indications for ICU admission were heterogeneous, which limits generalizability, as distinct underlying pathologies may variably affect S1P production, carrier availability (albumin and HDL), and downstream signalling. The observation window was short (day 0–3), potentially missing longer biological trajectories, and the cohort was not enriched for specific endotypes such as overt endothelial dysfunction or lymphopenia, in which albumin’s biological effects might be most pronounced.

#### Confounding and causal interpretation

Because treatment was clinician-guided, HA-treated patients were more severely ill at baseline (higher SOFA score; SAPS II not significantly different). This limits causal interpretation of between-group contrasts and justifies our focus on within-patient pre/post analyses. To mitigate confounding by indication, results were confirmed in adjusted models including SOFA, age, and sex, which yielded directionally consistent findings. Nevertheless, residual and time-varying confounding cannot be excluded.

#### Statistical sensitivity and exploratory nature

In the treated subgroup (*n* = 22), the pre/post increase in serum albumin from 1.8 g/dL (IQR 1.4–2.1) to 2.4 g/dL (IQR 2.1–3.3) represents a large and clinically meaningful biochemical change, confirming adequate sensitivity for the primary endpoint. However, the study was not powered to detect small-to-moderate effects, particularly in immune-cell subsets or subgroup analyses; these results should therefore be regarded as exploratory.

#### Mechanistic assays and analytical scope

The ECIS findings should be interpreted with caution. The absence of measurable effects likely reflects that most patients did not present with pronounced endothelial dysfunction at sampling. Mechanistically, our ex-vivo assays (ECIS and chemotaxis in native plasma) preserved the physiological milieu but cannot isolate carrier-specific contributions. The carrier-defined Ca²⁺ assay provided receptor-level confirmation at low-nanomolar S1P yet does not resolve kinetic or downstream-pathway differences. ApoM(+) HDL subfractions, HDL functionality, and S1P-handling enzymes or transporters (e.g., SPNS2, MFSD2B) were not analysed and may affect compartmental dynamics. In addition, ECIS experiments were performed in medium containing 2% fetal bovine serum, which provides baseline concentrations of albumin (~ 0.8 g/L) and HDL (~ 20 µg/L). This background presence of S1P carriers may have reduced the sensitivity to detect incremental carrier-dependent effects of patient plasma.

#### Co-interventions and unmeasured variables

Concomitant ICU therapies - including corticosteroids, vasopressors, and antibiotics – may influence S1P signaling, immune trafficking, and vascular permeability. Although some data were available, dosing and timing were heterogeneous and incomplete; to avoid model instability, these variables were not included as covariates. Time-dependent confounding from such co-interventions therefore cannot be ruled out.

#### Future directions

Taken together, these limitations highlight the need for larger, mechanistically focused studies that move beyond biochemical endpoints toward causal and translational understanding. Future investigations should prospectively standardize co-interventions and include endotype-enriched populations - particularly those with pro-inflammatory states, endothelial dysfunction, or low-HDL/low-ApoM profiles - in which albumin’s non-oncotic effects may be most pronounced. Mechanistic work integrating apoM-defined HDL fractions, broader concentration ranges, and kinetic or signalling readouts with in vivo validation will be essential to refine causal inference and contextual relevance.

From a translational perspective, it will also be important to evaluate combination strategies, such as HA administration alongside agents that target the S1P–HDL axis (e.g., statins), which may potentiate endothelial and immune stabilization. Such designs, coupled with adequately powered confirmatory cohorts, could ultimately determine whether albumin-mediated modulation of S1P carriage translates into measurable immunological benefit and improved outcomes in selected critically ill populations^45^.

## Conclusion

The clinical value of human albumin (HA) in critically ill patients remains debated. While randomized trials show inconclusive results, clinical experience suggests benefits beyond volume expansion, potentially linked to HA’s role as a carrier for sphingosine 1-phosphate (S1P), involved in endothelial stability and immune modulation. As summarized in Fig. 5, our study shows that HA does not negatively affect plasma S1P levels but redistributes S1P from HDL to serum albumin (SA), which seems to modulate immune responses. Ex vivo, HA had no direct impact on endothelial barrier function or cell migration, suggesting no negative effect on these parameters. However, these findings are difficult to interpret, as the inclusion criteria did not focus on endothelial dysfunction or lymphopenia, conditions where HA’s effects might be more evident. Future studies should explore whether specific criteria, such as low HDL or hyperinflammation – are more relevant for identifying patients who might benefit from HA’s immunomodulatory effects.

## Data Availability

The data that support the findings of this study are not openly available due to reasons of sensitivity and are available from the corresponding author upon reasonable request.

## Abbreviations

ALBIOS: Albumin Italian outcome sepsis study
ALT: Alanine aminotransferase
AST: Aspartate aminotransferase
CRP: C–reactive protein
EARSS: Early albumin resuscitation sepsis study
ECIS: Electric cell–substrate impedance sensing
EDTA: Ethylenediaminetetraacetic acid (anticoagulant for blood samples)
FACS: Fluorescence–activated cell sorting
FIDA: Flow–induced dispersion analysis
HA: Human albumin
HDL: High–density lipoprotein, HDL–cholesterol
HPLC: High–performance liquid chromatography
ICU: Intensive care unit
IL-6: Interleukin 6
LC/MS/MS: Liquid chromatography–tandem mass spectrometry
LDL: Low–density lipoprotein
LOS: Length of stay (hospitalization)
PBS: Phosphate–buffered saline
PCT: Procalcitonin
RCF: Relative centrifugal force
SAFE: Saline vs. albumin fluid evaluation
SAPS: Simplified acute physiology score
S1P: Sphingosine–1–phosphate
SOFA: Sequential organ failure assessment score
SOP: Standard operating procedure
SA: Serum albumin
